# On the Supposed Evidence for Libertarian Paternalism

**DOI:** 10.1007/s13164-015-0248-1

**Published:** 2015-05-17

**Authors:** Gerd Gigerenzer

**Affiliations:** Center for Adaptive Behavior and Cognition, Max Planck Institute for Human Development, Lentzeallee 94, 14195 Berlin, Germany

## Abstract

Can the general public learn to deal with risk and uncertainty, or do authorities need to steer people’s choices in the right direction? Libertarian paternalists argue that results from psychological research show that our reasoning is systematically flawed and that we are hardly educable because our cognitive biases resemble stable visual illusions. For that reason, they maintain, authorities who know what is best for us need to step in and steer our behavior with the help of “nudges.” Nudges are nothing new, but justifying them on the basis of a latent irrationality is. In this article, I analyze the scientific evidence presented for such a justification. It suffers from *narrow logical norms*, that is, a misunderstanding of the nature of rational thinking, and from a *confirmation bias*, that is, selective reporting of research. These two flaws focus the blame on individuals’ minds rather than on external causes, such as industries that spend billions to nudge people into unhealthy behavior. I conclude that the claim that we are hardly educable lacks evidence and forecloses the true alternative to nudging: teaching people to become risk savvy.

Liberty cannot be preserved without a general knowledge among the people …

John Adams, 1765

Bounded rationality is not irrationality. … On the contrary, I think there is plenty of evidence that people are generally quite rational; that is, they usually have reasons for what they do.

Herbert Simon 1985

Libertarian paternalism, as it is called, is a variant of soft paternalism that uses a bag of tricks called “nudges” to influence people’s decisions. A nudge is a tool for influencing people without using incentives, which are the lifeblood of economic theory, and without enforcing behavior, the essence of hard paternalism. The program is called “paternalistic” because it tries to guide people and “libertarian” because no choices are taken away. In the best paternalistic spirit, its goal is to protect others from harm. Yet its rationale is not to defend us from enemies outside. Instead, the program wants to protect us from ourselves, from our systematic reasoning errors, inertia, and intuition.

Nudging is nothing new. Governments and marketing agencies have relied on it for a long time. Consider the appointment letters sent in many countries to women above age 50 for mammography screening. These letters contain a preset time and location. This default booking is a nudge that exploits inertia—women might not take the effort to actively sign up and, similarly, not take the effort to decline the appointment. Furthermore, in the letters and pamphlets encouraging screening, it is often stated that early detection reduces breast cancer mortality by 20 %. That figure is a second nudge that exploits people’s statistical illiteracy. Screening reduces breast cancer mortality from about 5 to 4 in 1,000 women (after 10 years), which amounts to an *absolute* risk reduction of 1 in every 1,000. But this figure is typically presented as a *relative* risk reduction of 20 %, often rounded up to 30 %, to look more impressive (Gigerenzer 2014a, b).

This example illustrates the difference between nudging and educating. The aim of the appointment letters is to increase participation rates, not understanding. As a result, women in the European Union are less knowledgeable about the benefit of screening than Russian women, who are not nudged by relative risks and similar persuasive techniques (Gigerenzer et al. 2009). Education, by contrast, aims at “a general knowledge among the people” (see introductory epigram), and would require measures to make the public statistically literate and to enforce transparent information policies so that citizens can make informed decisions. But there are often conflicts of interest: in the case of mammography screening, informed citizens might understand that only few women benefit while many are harmed, which would likely decrease participation rates.

The example also serves to illustrate the difference between nudging and hard paternalism. Whereas women in Europe and the US can opt out, the president of Uruguay, an oncologist, made biennial screening mandatory for all working women age 40 to 59 (Arie 2013).

The interest in nudging as opposed to education should be understood against the specific political background in which it emerged. In the US, the public education system is largely considered a failure, and the government tries hard to find ways to steer large sections of the public who can barely read and write. Yet this situation does not apply everywhere.

## Nudging Is Not the Same as Libertarian Paternalism

In the literature, nudging and libertarian paternalism are conflated. Nudging is a label for non-coercive ways already known to steer people. The celebrated etched black fly in airport urinals to reduce spillage and the stripes on Chicago highways to slow down drivers are instance of nudges integrated into the design of everyday things (Norman 1990), based on J. J. Gibson’s concept of affordances. My own research program on ecological rationality is another point in case, where we teach laypeople and experts to use representations such as absolute risks and heuristic tools such as fast-and-frugal trees to make better decisions (Gigerenzer 2014a, b; Gigerenzer et al. 2011).

In this article, I do not argue against nudging per se. But I do object to the justification of such techniques on the basis of people’s lack of rationality by libertarian paternalists such as Thaler and Sunstein (2003, 2008). This justification focuses the blame for societal problems exclusively on the individual mind, closing our eyes to institutions that steer individual behavior so that they can take advantage of it, and it misleadingly suggests that a more sustainable solution, educating people, is a hopeless endeavor. Thus, the target of my analysis is the program of libertarian paternalism, not nudging per se.

A second obstacle to every fruitful discussion of nudging is the multiplicity of its meanings. Since the publication of Thaler and Sunstein’s (2008) *Nudge*, almost everything that affects behavior has been renamed a nudge, which renders this concept meaningless.^1^ Thus it is important to stick to its original meaning, which I define here consistently with the thoughtful analysis by Rebonato (2012):

Libertarian paternalism is a set of interventions aimed at overcoming people’s stable cognitive biases by exploiting them in such a way as to steer their decisions towards the choices they themselves would make if they were rational.

In the case of appointment letters, the biases targeted are inertia and lack of statistical literacy, and both are exploited to steer women into screening. The political philosophy of libertarian paternalism consists of three parts:

- *Objective*. A benevolent “choice architect” (policy maker) determines what is best for the people. The people themselves, however, are rarely asked because they are assumed to lack rationality (see justification).
- *Engineering*. A nudge is introduced to change people’s behavior in the desired direction, without incentives, coercion, or education.
- *Justification*. To justify why governments should nudge their citizens in the first place rather than educate them, libertarian paternalists call upon psychological research that has allegedly shown people’s systematic lack of rationality and inability to unlearn their errors.

In this article, I investigate the scientific evidence for the justification of nudging and the benevolent choice architect. I do not analyze the engineering part, given that the engineering tools were mostly known before the term *nudging* was even coined. Their impact was investigated by the House of Lords (2011), whose “central finding is that non-regulatory measures used in isolation, including ‘nudges’, are less likely to be effective” (p. 5). The report concluded that there “is a lack of applied research at a population level to support specific interventions to change the behavior of large groups of people (including lack of evidence on cost-effectiveness and long-term impact)” (p. 18). Missing proof of effectiveness *at the population level* need not be held against the nudging tools, I believe, given that so little funding is spent on behavioral factors—only 0.5 % of health research in the UK. But a danger is that nudging may become an excuse for not protecting consumers. The House of Lords criticized the Cameron Government for focusing on nudging citizens instead of considering other, more efficient options such as prohibiting television advertising of products high in fat, salt, and sugar. Before beginning, however, I would like to acknowledge that libertarian paternalists have made an impressive effort in making governmental officials aware of psychological factors.

In no way do I claim to be exhaustive in my analysis, but I hope to supplement the reader with that part of psychological evidence that has been left out of the standard justification for nudging. As I will argue in some detail, the dismal picture of human nature painted by behavioral economists and libertarian paternalists is *not* justified by psychological research. Rather, it is largely the product of narrow logical norms of rationality and selective reporting of the psychological literature. Most important for public policy, by comparing cognitive illusions with visual illusions, libertarian paternalists misleadingly suggest that attempts to liberate people from their biases through education are largely doomed to fail. However, as I will show, there is experimental evidence that even children can learn to deal with risk and uncertainty—if they are taught how. I will conclude that democratic governments should invest less in nudging and more in educating people to become risk savvy.

### The Enemy Within

According to libertarian paternalism, psychological research has discovered that people are not rational but suffer from cognitive illusions. The point is not simply that people make mistakes; that is nothing new. Their key point is that something inside our mind makes virtually everyone err in systematic ways. In Ariely’s (2008, p. xviii) words, the conclusion is that “we are not only irrational but *predictably irrational* – that our irrationality happens the same way, again and again.” What are these irrationalities? Thaler and Sunstein (2003) explain:

“People do not exhibit rational expectations, fail to make forecasts that are consistent with Bayes’ rule, use heuristics that lead them to make systematic blunders, exhibit preference reversals (that is, they prefer A to B and B to A) and make different choices depending on the wording of the problem” (p. 176).

In this account, the enemy is within us, embodied in the very nature of our thinking. As Thaler and Sunstein (2008) wittily asserted, humans are not even remotely like Homo economicus, but more like *Homer Simpson*. That message has become extremely popular, precisely because it is directed against neoclassical economists and other libertarians. For instance, in her book *Against Autonomy* (2013), legal philosopher Sarah Conly concluded that John Stuart Mill “failed to adequately reckon with human psychology, as we now know it to be” (p. 8) and that “the existence of cognitive deficits does suggest a need for different sorts of legislation, […] coercive paternalism, for laws that force people to do what is good for them” (p. 2–3). In his essay “Paternalism and Cognitive Bias” (2005), philosopher J. D. Trout maintained: “Our review of the biases will show that they are virtually as stable, durable, and universal as reflexes” (p. 396) and “that the Enlightenment vision is profoundly mistaken” (p. 397). Likewise, behavioral economist Richard Thaler (1991) asserted that “mental illusions should be considered the rule rather than the exception” (p. 4). With somewhat more nuance, psychologist Daniel Kahneman (2011) stated: “Democracy is inevitably messy, in part because the availability and affect heuristics that guide citizens’ beliefs and attitudes are inevitably biased, even if they generally point in the right direction” (p. 145).

In spite of this heavy-duty rhetoric, libertarian paternalists do not try to overthrow Homo economics. On the contrary, as the quoted list of irrationalities illustrates, they rather uncritically accept the rules of axiomatic decision theory as the *norm* for all rational behavior, and blame mortals for not living up to this ideal. As a result, *Homo sapiens* (“man the wise”) appears to be a misnomer, and on two counts:

- *People Lack Rationality* The claim is that experiments have unambiguously shown that people suffer from systematic reasoning errors, due to their cognitive limitations.
- *People Are Hardly Educable* The claim that people largely cannot unlearn these errors is typically made through the use of analogies rather than evidence. The three analogies used are *visual illusions*, the *reptilian brain*, and a biologically hard-wired *System 1* that relies on heuristics and intuition rather than on statistics and logic. The choice of analogies aligns cognitive errors with biological determinism. For instance, comparing reasoning errors—aptly called *cognitive illusions*—to visual illusions implies that trying to educate people out of them is a doomed effort.

In short, the argument is that cognitive deficits are both widespread and next to impossible to eradicate. The second claim is not always stated explicitly, but without it, paternalists would have to explain why they prefer nudging to educating people. These two claims provide the rationale for governmental intervention. Libertarian paternalism’s justification for intervention is quite different from that of neoclassical economic theory, where intervention may be deemed necessary in cases of imperfections of the market, such as when a firm has a monopoly, or when free markets do not produce a fair distribution of income. To redress these imperfections or inequalities, governments can interfere. However, if, as libertarian paternalists say, the imperfections are engraved in our brains rather than in the market, there is little hope of redressing them. In this very sense, libertarian paternalism is more red-blooded than some forms of hard paternalism, even if it does not use coercion (Rebonato 2012). Hard paternalists may justify intervention on the grounds that individuals rationally pursue their selfish goals instead of the welfare of the society. Libertarian paternalists, in contrast, advocate that people do not know how to pursue their own goals and may not even know what goals are worth pursuing in the first place.

In this article, I will argue:

1. The evidence for systematic irrationality is far from being as clear-cut as claimed by libertarian paternalists. Specifically, their claims have been based on (i) *narrow logical norms*, that is, a misunderstanding of the *ecological* nature of rationality, and (ii) a *confirmation bias*, that is, selective reporting of research.
2. Nor is the argument that people are non-educable backed by clear-cut empirical evidence. Instead, studies show that children, adults, and experts can learn statistical thinking with the help of adequate numerical or visual representations. Educating people to become more risk savvy is the true alternative to the program of blaming and nudging people (Gigerenzer 2014a, b).
3. Libertarian paternalism requires a technocracy of experts who know what is best for us in order to steer us there. This assumes choice architects who do not suffer from the same cognitive errors and who pursue no conflicting interests. Among professionals in health and wealth, however, studies show that such benevolent professionals and governmental officials are largely nonexistent. The remedy is the same as in Point 2: to invest in educating people to become risk savvy so that they can critically evaluate governmental policies.
  These three points bring to light the individualistic bias that the libertarian paternalism program inherited from its intellectual source, the heuristics-and-biases program (e.g., Kahneman and Tversky 1972). Virtually every blunder is attributed to a flaw in the human mind, even if part of the problem lies in industries that persuade individuals into unhealthy behaviors, from smoking to fast food to excessive alcohol consumption. As we will see, strategic interaction and social intelligence are mistaken for logical error (Point 1), errors are attributed to lack of rationality rather than to lack of education (Point 2), and choice architects are assumed to be benevolent philosopher-kings rather than employees of organizations that may pursue conflicting interests (Point 3).

## On the Evidence for Systematic Deviations from Rationality

I will restrict my analysis to three of the allegedly stable cognitive errors cited by Thaler and Sunstein (2003, p. 176) above:

1. People “make different choices depending on the wording of the problem,” which is known as the *framing effect*.
2. People “fail to make forecasts that are consistent with Bayes’ rule,” which is known as the *base rate fallacy*.
3. People “use heuristics that lead them to make systematic blunders,” which is part of the postulate that using statistics and logic always leads to more accurate judgments than when relying on heuristics and intuition.
  To be clear, I will evaluate the very experimental evidence that libertarian paternalists present to make their case for systematic human irrationality. I am not arguing that people never make errors (nor would neoclassical economists make such an argument). There are multiple other reasons for harmful behavior, including the fast food and tobacco industry’s multi-billion-dollar advertisement campaigns to seduce people into unhealthy lifestyles and the failure of educational systems worldwide to teach children statistical thinking. Libertarian paternalists, like the behavioral economists they draw upon, make a case against the human mind and thereby turn a blind eye to flaws in human institutions.

### Framing Effects

A framing effect occurs when people’s choices differ depending on how two “logically equivalent” statements are framed. This variation is said to be inconsistent with rational behavior because it violates *description invariance*, an “essential condition for a theory of choice that claims normative status” (Tversky and Kahneman 1986, p. S253).

Framing effects are of central importance for libertarian paternalism. They justify the argument that there is no viable alternative to paternalism: Because it is impossible to avoid framing options, someone must decide how to do it (Thaler and Sunstein 2008, p. 11). Framing effects also justify why paternalists rarely try to find out what preferences individuals actually have, in spite of emphasizing that a “policy is ‘paternalistic’ if it tries to influence choices in a way that will make choosers better off, *as judged by themselves*.” (p. 5; emphasis in the original). The justification is that people are unreliable sources—their answers and “revealed preferences” may depend on how the question is framed (Thaler and Sunstein 2003, p. 178). In this view, it is unfruitful to ask people, subject to these biases, what they really want. In Bazerman and Neale’s (1986, p. 317) words, framing effects “suggest that individuals are generally affected by systematic deviations from rationality.”

I disagree with the assertion that logical equivalence or description invariance constitutes a *general norm* for rational behavior. Here is why.

Consider the prototype of all framing examples:

- The glass is half full.
- The glass is half empty.

Should the choice of description matter? In an experiment, a full glass of water (A) and an empty one (B) are put on the table. The experimenter asks the participant to pour half of the water into the other glass, and then to place the “half-empty glass” at the edge of the table. Most people choose glass A, the previously full glass (Sher and McKenzie 2006). This and similar experiments show that in many situations, the framing of a request encodes *surplus* information, here about the past state of the glass, that serves as a *reference point*, and that most people intuitively understand which glass is meant (McKenzie and Nelson 2003). There is nothing irrational about social intelligence, which here entails the ability to listen carefully to a speaker and reduce uncertainty. At issue are pragmatic inferences, not logical ones. More generally, the analysis of the relation between mind and environment—here between speaker and listener—is called the study of *ecological rationality* (Gigerenzer and Selten 2001). Description equivalence, in contrast, is a form of logical rationality. As a normative theory of choice, logical rationality does not enable us to decipher the message.

Now consider a case of framing presented by libertarian paternalists (Sunstein 2013, p. 61; Thaler and Sunstein 2008, p. 39). You suffer from a serious heart disease, consider a dangerous heart surgery, and ask your doctor about its prospect. The doctor can frame the answer in two different ways:

- Five years after surgery, 90 % of patients are alive.
- Five years after surgery, 10 % of patients are dead.

Thaler and Sunstein (2008) argued that you should *not* pay attention to how your doctor frames the message. Citing a single study, they asserted that in numerous experiments people react very differently “even though the content of the two statements is exactly the same” (p. 39) and concluded that “framing works because people tend to be somewhat mindless, passive decision makers” and so it offers a “brief glimpse at human fallibility” (p. 40).

Before we go on, let us briefly consider the actual evidence. A systematic review of 40 framing studies with mostly hypothetical choices showed that participants were indeed more likely to consent to surgery in the survival frame than in the mortality frame. The review also showed that this effect was specific rather general: There was no evidence of a framing effect for consent to medication or immunization instead of surgery, nor when real as opposed to hypothetical decisions were studied (Moxey et al. 2003).

Now let us apply the same ecological analysis as with the half-full/half-empty glass to the survival/mortality frame in the surgery scenario. Instead of regarding the logical structure alone, consider the patient’s goal. To make a rational decision, the patient needs to know the answer to the question “Is survival higher with or without surgery?” Neither “90 % survival” nor “10 % mortality” provides sufficient information for a rational decision. What the patient also needs to know is the survival rate *without* surgery. Although that is the reference point against which the surgery option needs to be compared, this essential information is not explicitly stated in this textbook problem, where there is no doctor to ask. Thus, participants have to rely on their social intelligence to make an informed guess.

By framing the option, speakers can convey information about the reference point, something listeners tend to intuitively understand. Experiments showed that if the reference point was lower (“fewer patients survive without surgery”), then 80–94 % of the participants chose the “survival” frame, but if the reference point was higher (“more patients survive without surgery”), then the survival frame was chosen less frequently (McKenzie and Nelson 2003). Thus, a doctor’s choice between logically equivalent frames can provide relevant information about the reference point. By choosing a survival frame, the doctor can communicate that surgery has a substantial benefit compared to no surgery, and make an implicit recommendation. To conclude, logically equivalent frames are not necessarily informationally equivalent.

Note that the reference point that is implicitly communicated by framing an option has nothing to do with prospect theory’s reference point. Prospect theory is based on an individualistic analysis that pays no attention to strategic interaction.

To illustrate the generality of the argument against logical norms, consider a final example, the notorious Asian disease problem (Tversky and Kahneman 1981), where a disease is expected to kill 600 people and participants have to choose between two programs. The benefits of the programs are framed positively or negatively. Here is the positive (gain) frame:

If Program A is adopted, 200 people will be saved (*riskless option*).

If Program B is adopted, there is a one-third probability that 600 people will be saved and a two-thirds probability that no people will be saved (*risky option*)

And here the negative (loss) frame:

If Program C is adopted, 400 people will die (*riskless option*).

If Program D is adopted, there is a one-third probability that nobody will die and a two-thirds probability that 600 people will die (*risky option*)

In comparison with the previous two framing tasks, now a risky option is added. Many experiments showed that when choosing between gains, a majority preferred the riskless option A, but when choosing between losses, a majority preferred the risky option D. Once again, the positive frame is said to be logically equivalent to the negative frame, which makes the majority choice appear logically inconsistent.

But look carefully at the wording, and you will notice something. The risky options are completely specified, whereas the riskless options are not. For instance, the riskless option notes that “200 people will be saved,” without adding “and 400 will not be saved.” That should make no difference according to logical rationality, given that framing in terms of gains and losses is preserved. Nor should it make a difference from the logic of prospect theory. But it should make a difference for intelligent people, because incomplete specification is the very tool for making implicit recommendations. This is easy to see after noticing that the two (riskless) options that are only partially specified correspond to the two frames of the surgery problem. Consistent with this ecological rather than logical analysis, studies reported that the framing effect is driven by the riskless options, *not* by the risky options (see Kühberger and Gradl 2013). Moreover, when researchers completely specified the riskless options, the framing effect in the Asian disease problem disappeared (Kühberger 1995; Kühberger and Tanner 2010; Mandel 2001). Once again, this suggests that people tend to assume that the choice of frame “leaks” information, that is, makes an implicit recommendation. If this possibility is eliminated by adding the unspoken alternative, then the framing effect disappears.

In sum, research on these three classical framing effects indicates that logical equivalence is a poor general norm for understanding human rationality. An ecological analysis suggests that speakers rely on framing in order to implicitly convey relevant information and make recommendations, and that listeners also pay attention to these. In these situations, framing effects clearly do not demonstrate that people are “mindless, passive decision makers,” as Thaler and Sunstein (see above) asserted. In fact, the art of reading between the lines is more cognitively demanding than the narrow logic of descriptive invariance. After all, computers have no problems mastering logic but still struggle with understanding natural language.

As mentioned above, libertarian paternalists do not see any alternative to nudging, given that a message is always framed in some form or another. Yet the previous examples show that *there is indeed a simple alternative* to positive or negative framing, namely to specify a quantitative message in its entirety, such as “90 % of patients are alive and 10 % are dead.”

#### Strategic Interaction Mistaken as Logical Error

The previous analysis of framing effects in terms of communicating reference points and signaling recommendations does not apply to all framing effects. But it should suffice to make the point that descriptive invariance is not a reasonable yardstick for rational behavior in general. The same analysis applies to various other alleged cognitive illusions, such as default effects and the Linda problem. Whereas paternalists tend to explain default effects, as in organ donation, by people’s inertia, Johnson and Goldstein (2003) found substantial default effects even when no effort was required, and subsequent experiments indicated that many people interpret policy makers’ choice of default as the recommended action (McKenzie, Liersch, and Finkelstein 2006). Similarly, although Thaler and Sunstein (2008, p. 29) called people’s majority response in the Linda problem “an obvious logical mistake,” other research concluded that what they assumed to be a logical error is in fact social intelligence at work (once again). This research has also shown how to make the “error” largely disappear (e.g., Fiedler 1988; Hertwig and Gigerenzer 1999; consistent with earlier research on set inclusion by Inhelder and Piaget (1964, p. 101). Finally, a systematic review of hundreds of framing studies could not find a single one showing that framing effects incur real costs in terms of health or wealth (Arkes, Gigerenzer, and Hertwig 2015).

Libertarian paternalists and some behavioral economists may be among the last professionals who cherish the ideal that logic by itself provides a universal yardstick for human intelligence. Even neoclassical economists have gone beyond logical rationality; the ecological analysis provided above is akin to research on signaling, co-ordination games, and strategic interaction. For instance, Sen (1993) argued that the rationality of behavior cannot be determined by using a purely logical principle such as consistency without investigating people’s goals and motives. Even when there is no strategic interaction, the idea that logically equivalent formulations should not matter misses the essence of real intelligence. The physicist Richard Feynman (1967) was keen to point out the importance of different formulations of the same physical law, even if they are logically equivalent: “Psychologically they are different because they are completely unequivalent when you are trying to guess new laws” (p. 53). Logic is certainly a tool of rationality, but is not the only one in the toolbox.

All in all, the principle of descriptive invariance is, by itself, an inappropriate yardstick of rationality. Framing effects, defined as the violation of this principle, can be the result of strategic interaction, interpreted signaling of recommended options, and other forms of social intelligence. These typically intuitive forms of intelligence are misinterpreted in the behavioral economic literature as cognitive errors that are hard to unlearn. What this literature overlooks is that when intuition is smarter than logic, there is little need to educate people out of their “logical errors.”

#### Paternalists’ Confirmation Bias

I have devoted some space to studies that question the logical norm of descriptive invariance because these studies are rarely if ever discussed by libertarian paternalists, despite the importance they attribute to framing effects. You can call this a bias for people’s biases, or a confirmation bias: presenting studies that appear to demonstrate people’s systematic deviations from rationality but omitting those from researchers who do not find biases or disagree with the yardstick of rationality used in the first place. For instance, to my knowledge the well-known research of the McKenzie group on framing has been mentioned neither in the libertarian paternalist literature nor in most of the behavioral economics literature that argues that all framing effects violate rationality. I have observed the same confirmation bias for other so-called cognitive illusions (see Gigerenzer, Fiedler, and Olsson 2012; Gigerenzer 2000). Here is one last example.

“One of the most significant and irrefutable findings of behavioral psychologists is that people are overconfident in their judgments” (Parikh 2009, p. 142). Similar absolute statements are frequent, indicating high confidence in overconfidence. A glance into the psychological research, however, shows a quite different picture. To begin with, half a dozen different phenomena whose relation is currently unknown have been labeled overconfidence. Consider first *miscalibration of subjective probabilities*, one of these allegedly irrefutable findings. Since the mid-1990s, however, studies have shown that what is called miscalibration is not a systematic error by ordinary people, as claimed, but an artifact—that is, a systematic error made by researchers who mistake people’s *unsystematic* error for a systematic one (details in Dawes and Mulford 1996; Erev et al. 1994). This is known as misinterpreting regression to the mean, which is a consequence of an imperfect correlation, not of systematic bias. The same error appears to have made in the classical study allegedly demonstrating that people systematically overestimate small risks and underestimate large risks (Slovic, Fischhoff, and Lichtenstein 1982). A reanalysis showed that the “miscalibration” is largely a result of regression to the mean and not due to systematic biases of the participants, as was and still is reported (Hertwig, Pachur, and Kurzenhäuser 2005). A second phenomenon that is also called overconfidence (mean confidence minus proportion correct) has been shown to be due to researchers’ unrepresentative (i.e., selective) sampling of questions: An analysis of 130 studies showed that overconfidence disappears when questions are representatively sampled (Juslin, Winman, and Olsson 2000). These ecological analyses should suffice to make the point; for more on overconfidence research, see Olsson (2014).

This is not to say that people are never overconfident. If you earn your money by forecasting exchange rates or the stock market, you had better be overconfident; otherwise no one will buy your advice. But functional overconfidence is not the same as people’s hardwired cognitive illusion. My point here is different. In the paternalist literature I have not seen discussions of the existing research that concludes that most people have unsystematic, but not systematic biases. Nor have I seen references to the research concluding that some paternalists’ claims are based on their own errors in statistical thinking, as illustrated above. Based on analyses of hundreds of studies on overconfidence, evidence for both of these conclusions has been compiled by the group around Peter Juslin (e.g., Juslin, Winman, and Olsson 2000; Juslin, Winman, and Hansson 2007). Yet this research is ignored in the nudging literature.

### Bayes’ Rule

Thaler and Sunstein (2008) argued that people “fail to make forecasts that are consistent with Bayes’ rule” (cited above). This claim goes back to Kahneman and Tversky (1972, p. 450), who rejected earlier research concluding that people are approximate, albeit conservative Bayesians: “In his evaluation of evidence, man is apparently not a conservative Bayesian: he is not Bayesian at all.” Unlike descriptive invariance, Bayes’ rule is a strict consequence of the axioms of probability. Thus, here we have a real test case for rationality. If the conditions for Bayes’ rule are met, and if people’s forecasts systematically deviate from the rule and they do not learn from errors, then there is a good case to be made that they are behaving irrationally. I cannot review here the vast psychological literature on Bayesian reasoning, but will simply point out those parts that arrived at strikingly different conclusions than those suggested by the libertarian paternalists.

There are two research paradigms for studying Bayesian inference, the probability learning paradigm and the text problem paradigm. In the learning paradigm, people learn probabilities from experience, and are subsequently tested as to whether they make judgments consistent with Bayes’ rule. This research is methodologically similar to the revealed preferences approach in economics. Its results contradict the unqualified assertion that people fail to make forecasts consistent with Bayes’ rule. On the contrary, many cognitive scientists conclude that people’s judgments are largely consistent with it (e.g., Chater, Tenenbaum, and Yuille 2006; Chater and Oaksford 2008; Edwards 1968). Consider Anderson’s (1990) *rational analysis* program, a term chosen “out of analogy to the rational man approach in economics” (p. x). This ecological approach models both conscious processes such as causal inference and problem solving *and* unconscious processes such as memory and forgetting as Bayesian inference (Schooler and Anderson 1997). Similarly, in “Bayesian Models of Cognition,” Griffiths, Kemp, and Tenenbaum (2008) argued that both unconscious perceptual processes, such as inferring the color and shape of objects, and high-level cognition, such as language understanding and categorization, are consistent with Bayesian models. As a final case, there is an influential Bayesian program in neuroscience that assumes a “Bayesian brain” (e.g., Friston 2010).

Behavioral economists routinely claim that fast, unconscious, and automatic judgments (the so-called “System 1”) do *not* work by the rules of probability. Yet, according to the many cognitive scientists just mentioned, they do.

The second paradigm does not involve probability learning. Instead, researchers confront participants with text problems in which probabilities are numerically stated. These tasks are also called *decisions from description* as opposed to *decisions from experience* (Hertwig and Erev 2009). Within this paradigm are two kinds of text problems. In the first, the only numbers participants are given are the base rates, such as that 30 engineers and 70 lawyers were interviewed. Then they are given the description of a person, such as one that incorporates stereotypical traits of an engineer. On average, people’s estimates of the probability that the person was an engineer were for the most part the same, independent of whether there were more engineers or lawyers (Kahneman and Tversky 1973). This and similar results were interpreted as instances of the base rate fallacy and hailed as “perhaps one of the most significant departures of intuition from the normative theory of prediction” (p. 243).

As every practicing statistician knows, however, there is more to rationality than inserting numbers into Bayes’ rule. There are assumptions that need to be checked. A crucial assumption for the relevance of the base rates is that the person has been randomly sampled from the population with the base rates specified. If not, the normative relevance of the base rates is not given. Nonetheless, in some text problems, including the celebrated “Tom W.” problem (Kahneman and Tversky 1973), *no* information was given about random sampling. In others, such as the engineer-lawyer problem, random sampling was asserted, but wrongly so, because the descriptions were fabricated and not randomly sampled (Gigerenzer 2000, Chapter 12). In fact, when the engineer-lawyer experiment was replicated so that people could randomly draw a description out of an urn, their neglect of base rates largely disappeared (Baratgin and Noveck 2000; Gigerenzer, Hell, and Blank 1988). Thus, one might tentatively conclude that ordinary people are more sensitive to the assumption of random sampling than the researchers who accuse them of irrationality.

The second kind of textbook problems provides not only the base rate but also the hit rate and the false alarm rate, and participants are asked to estimate the posterior probability. An example is the “cab problem” (Tversky and Kahneman 1980). In keeping with Thaler and Sunstein, there is robust evidence in this task that most people fail to make forecasts consistent with Bayes’ rule. But there is an important caveat: People fail to do so *if given the information in terms of conditional probabilities* (such as hit rates and false alarm rates). The reason for the failure is not simply in the human mind, but also in the ecology, that is, in how the information is presented. Most of us have difficulties with conditional probabilities.

If the information is presented as the outcome of learning from experience, known as *natural frequencies*, the proportion of people who reason in accord with Bayes’ rule rises substantially (Brase 2007, 2009; Gigerenzer and Hoffrage 1995; Hoffrage et al. 2000; Kleiter 1994). That holds for both laypeople and professionals. For instance, on the basis of conditional probabilities, only 21 % of 160 gynecologists correctly inferred the posterior probability that a woman has breast cancer if she had a positive screening mammogram. After the gynecologists were trained to translate conditional probabilities into natural frequencies, 87 % could derive the Bayesian posterior probability (Gigerenzer et al. 2007). A Cochrane Systematic Review (Aki et al. 2011) also concluded that health professionals and consumers understood natural frequencies better than probabilities. The same technique helps judges, attorneys, and law students understand what a DNA match means (Lindsey, Hertwig, and Gigerenzer 2003; Hoffrage et al. 2000), particularly when pictorial representations are used (Cosmides and Tooby 1996; Shapira et al. 2011; Spiegelhalter, Pearson, and Short 2011).

In sum, if one looks at the entire psychological literature, the claim that people generally fail to reason the Bayesian way is not supported by the evidence. Rather, an ecological analysis shows that certain presentations of information, such as natural frequencies, enable humans to reason the Bayesian way and that others prevent Bayesian thinking—just as we can do division more easily with Arabic numbers than with Roman ones. As mentioned above, the cognitive science research concluding that fast, intuitive inferences are consistent with Bayes’ rule directly contradicts the infamous “System 1” story that unconscious and elementary processes do not work by the rules of probability (see Sunstein 2013, 2014; Thaler and Sunstein 2008).

#### Can People Learn Bayesian Inference?

Are people indeed virtually ineducable, as the analogy between cognitive and visual illusions suggests? The study with gynecologists shows the opposite: Most doctors can learn to reason the Bayesian way in one CME session. But do people retain what they learn? A study entitled “Teaching Bayesian reasoning in less than 2 h” (Sedlmeier and Gigerenzer 2001) showed that 3 months after training with natural frequencies, there was no sign of the usual forgetting that occurrs when people learn to insert probabilities into Bayes’ rule. Even children can make forecasts consistent with Bayes’ rule. In a study in Beijing, 135 children were given more than 1,000 Bayesian problems framed in natural frequencies. The majority of 6th graders could judge the Bayesian posterior probability precisely, as could one third of 5th graders and one sixth of 4th graders (Zhu and Gigerenzer 2006). Not surprisingly, when the information was presented in conditional probabilities, not a single child could solve any of the problems. With the additional use of icons, German 4th graders could solve 60 % of Bayesian problems, and even 2nd graders could solve 22 % of them (Gigerenzer 2014a, 2014b; Multmeier 2012; see also Till 2013). Natural frequencies are now taught in mathematics curricula in German schools and their use is recommended by major evidence-based medical societies, such as the International Patient Decision Aid Standards Collaboration and the Medicine and Healthcare Products Regulatory Agency (the United Kingdom’s equivalent to the Food and Drug Administration).

All in all, the claim that people are poor Bayesians and can hardly unlearn their biases is not supported by evidence.

### Heuristics

Heuristics have gained an important role among libertarian paternalists, but mainly as being the cause of mental biases. People “use heuristics that lead them to make systematic blunders” (Thaler and Sunstein 2008, p. 176). By relying on logic or statistics rather than on heuristics, people would behave optimally, it is said. Yet the argument that rules of logic or statistics always define the optimal behavior is correct only in situations of risk, not of uncertainty (Binmore 2008; Knight 1921). The term *risk* refers to situations where all alternatives, consequences, and probabilities are known for certain, as in the Bayesian textbook problems mentioned above or when playing the roulette wheel in a casino. Here, one can calculate the expected loss in the long run, and no heuristics are needed. The term *uncertainty* refers to real-world situations where not everything is known, where surprises happen, and where there is no way to determine the optimal behavior, as in investment banking and much of health care. Here, rational decisions can be made by appropriate heuristics that aim at robustness rather than optimality. For Savage (1954), the father of modern Bayesian optimization theory, even planning a picnic, not to speak of playing chess, lies outside of Bayesian theory. This essential distinction between risk and uncertainty is missed by behavioral economists who assert that heuristics are always second best.

For instance, Thaler and Sunstein (2008, p. 133–136) reported that Harry Markowitz did not use his Nobel Prize-winning mean-variance portfolio when investing in his own retirement account, relying instead on the 1/*N* heuristic. This simple rule divides the available asset equally across the *N* options or stocks, without having to estimate the many parameters in the mean-variance portfolio. Comparing Markowitz to Homo Simpson in this case would be highly inappropriate, seeing as Markowitz himself discovered the method. DeMiguel, Garlappi, and Uppal (2009) tested how well 1/*N* did in the uncertain world of stocks. In six out of seven investment situations, 1/*N* outperformed the “optimal” mean-variance portfolio according to standard criteria, including the Sharpe Ratio. In this situation under uncertainty, making the optimization calculations can mean losing money. The financial crisis beginning in 2007 has opened the eyes of many to the fact that the standard probability models used, from value-at-risk to the Gaussian copula function, were part of the problem rather than the solution.

The real question is one of ecological rationality: to specify the environmental structures in which a given heuristic is likely more accurate than competing methods. For instance, 1/*N* is likely to result in better performance than Markowitz optimization when (i) the market is highly unstable, (ii) the number of options is large, and (iii) the sample size is small. Such analysis can explain inconsistent results when comparing 1/*N* with mean-variance portfolios (Brodie, Daubechies, De Mol, Giannone, and Loris 2009). The study of ecological rationality is described in Gigerenzer, Todd, and the ABC Research Group (1999), Gigerenzer, Hertwig, and Pachur (2011), and Gigerenzer and Selten (2001). It is a mathematical analysis that addresses Herbert Simon’s analogy of the “pair of scissors,” that is, how cognition and environment together produce rational behavior. Recall that Kahneman and Tversky have often acknowledged that heuristics are sometimes good and sometimes bad but have never gone a step further to specify when exactly “sometimes” is. The study of the ecological rationality takes that step. It contradicts the bare-bone claim that statistical optimization is always better than heuristics.

Finally, behavioral economists have made the general argument that violations of rationality axioms (such as when people rely on heuristics) provide a new rationale for paternalism. However, the argument that individual biases imply aggregate losses in efficiency and thus justify paternalism can be formally proven wrong (Berg and Gigerenzer 2007). “Bounded rationality” does not imply paternalism.

## On the Argument that People Are Not Easily Educable

As mentioned before, this argument is made implicitly by comparing cognitive illusions to stable visual illusions, the reptilian brain, and the supposedly biologically old “System 1” that is “not readily educable” (Kahneman 2011, p. 417). Evolutionary biologist Stephen Jay Gould (1992) similarly argued that “our minds are not built (for whatever reason) to work by the rules of probability” (p. 469), and Richard Thaler is quoted in a *Nature* article covering the debate on nudging versus educating as saying: “Our ability to de-bias people is quite limited” (Bond 2009, p. 1191). As a consequence, legal scholar Dan Kahan is quoted in the same article as saying, “Risk decision-making should be concentrated to an even greater extent in politically insulated expert agencies” (pp. 1189–1190).

I have two responses. First, consider situations of uncertainty (as opposed to risk), where the optimal action cannot be known. Here, paying attention to framing and using smart heuristics can be more intelligent than logical rationality. In such situations, there is little reason to educate people out of their intuitive intelligence into logical but inferior behavior. Second, consider situations of risk where statistical thinking is rational, but where people fail to reason according to some rule. In these situations, people can learn. That may require teaching them proper tools, such as natural frequencies. Back in 1986, Fong, Krantz, & Nisbett taught 347 adults and high school students from New Jersey suburban communities the law of large numbers. The 25 min training session had a marked effect on the quality of the participants’ reasoning about everyday problems. Similarly, Nisbett (2009) showed how IQ can be substantially improved by training. Teaching statistical thinking is a viable option, as anyone who attends the International Conferences on Teaching Statistics experiences.

The true alternative to nudging is education: making children and adults risk savvy. That encompasses statistical thinking and heuristic thinking, and judgments about the limits and possibilities of both approaches. Unfortunately, children in most countries are not taught statistical and heuristic thinking, the mathematics of uncertainty, but only the mathematics of certainty, such as geometry and trigonometry. Education, however, is outside the scope of libertarian paternalists. And that omission is not accidental but a direct consequence of a philosophy that compares cognitive errors to stable, inevitable visual illusions.

Yet that view ignores the fact that education is clearly associated with healthy behavior. Obesity, for instance, is more prevalent among less-educated people. Consider cancer, one of the most dreaded diseases. As we have seen, people are “nudged” into cancer screening, even when it saves few or no lives and instead harms many through unnecessary biopsies or surgery (Welch 2004). Education would be the better weapon because about half of cancers are due to behavior: mostly smoking, obesity, lack of movement, and alcohol. But to be effective, teaching would need to start early, before eating, drinking, and smoking habits are established in puberty. School programs that teach children the joy of cooking, the basics of a healthy life, and how to see through industrial attempts to seduce them into unhealthy behavior could save substantially more lives from cancer than screening and cancer drugs together (Gigerenzer 2014a, b). And such an early education can produce spillover effects to health in general.

## On the Assumption of Benevolent Choice Architects

Nudging people into what is best for them requires choice architects who actually know what is best for others. Yet this requirement contains an unresolved contradiction (Berg and Gigerenzer 2010; Rebonato 2012). On the one hand, experts are said to be subject to the same cognitive biases as ordinary people; on the other hand, they are supposed to be rational and discern what people really want or need. For instance, although Thaler and Sunstein have jokingly reported falling prey themselves to biases, they continue to write as if ultimately transcending these.

Moreover, the ideal of choice architects who act like benevolent philosopher-kings goes beyond merely discerning people’s true needs and preferences. It requires that choice architects:

1. do not practice defensive decision-making,
2. understand scientific evidence, and
3. have no conflicting interests.

Consider health care, where libertarian paternalists have argued that governments, hospitals, and doctors should nudge people into proper behavior. Such nudges would benefit patients only if these three conditions are in place. In health care in the US, Germany, and other Western countries, however, these conditions are typically not met (Gigerenzer and Muir Gray 2011).

1. *Self*-*defense*. Many health care providers practice defensive medicine. That is, instead of recommending what they believe is best for the patient, they suggest a second-best option in order to protect themselves from potential lawsuits. For instance, 93 % of 824 doctors in Pennsylvania admitted practicing defensive medicine, such as ordering unnecessary CT scans, antibiotics, and invasive surgery (Studdert et al. 2005).
2. *Innumeracy*. Many health care providers do not understand health statistics. For instance, 70–80 % of U.S. primary care physicians did not understand survival rates in cancer screening and could easily be manipulated into recommending screening, even if its harms exceeded its benefits (Wegwarth, Schwartz, Woloshin, Gaissmaier, and Gigerenzer 2012).
3. *Conflicts of Interest*. Some health care providers pursue profit instead of best practice. This state of affairs is a principal-agent problem in which the agent (doctor, hospital) is motivated to act in its own best interests rather than in those of the principal (patient). For instance, every year, an estimated one million U.S. children have unnecessary CT scans (Brenner and Hall 2007). A CT scan is a major source of income but exposes a child to radiation levels in the order of one hundred chest X-rays, contributing to an estimated 29,000 cancers that result from the approximately 70 million CT scans performed annually in the U. S., with figures rising (Berrington de González et al. 2009).

This trio of systemic biases has been dubbed the SIC *Syndrome* (Self-defense, Innumeracy, and Conflicts of interest) in health care (Gigerenzer 2014b). To the degree that the syndrome is in place, choice architects may steer the public into directions that are not in their best interest.

### When Choice Architects Pursue Objectives Against People’s Best Interests

#### Breast Self-Examination

Some choice architects have argued that women who do not perform monthly breast self-examination are “risk-averse” and have recommended using a loss frame instead of a gain frame to persuade women to do the exams (Meyerowtiz and Chaiken 1987; Salovey and Williams-Piehota 2004). Another nudging technique is to tell women that most other women their age regularly do self-examinations. Yet randomized trials found no evidence that self-examination actually reduces breast cancer mortality; instead there was evidence that it causes harms, such as an increased number of biopsies (Kösters and Gøtzsche 2003). To this day, many women are steered into self-examination without being informed that the scientific evidence shows no benefit. As mentioned above, health organizations nudge women into breast cancer screening by sending invitations without mentioning that randomized studies with over 500,000 women have failed to provide evidence that the benefits are larger than the harms (Gøtzsche and Jørgensen 2013). Here, nudging serves the interests of the multibillion mammography industry and does not enable women to make informed decisions.

#### Vaccination Against H1N1

In 2009, with the outbreak of the swine flu pandemic, a number of governments bought flu vaccines and medication such as Tamiflu, as recommended by the WHO. Yet uptake rates were low in some countries. Sunstein (2013, p. 59) suggested that compliance may be nudged by, for instance, giving people maps showing the route to the local vaccination center. That would indeed be a good tool when vaccination or medications actually reduce serious complications and death. But in the case of Tamiflu, years later there is still no evidence that it does. Roche, its manufacturer, refuses to reveal the data, despite requests by the *British Medical Journal* (which has published its correspondence with Roche on its website). Why did the WHO encourage governments in the first place to stockpile antivirals, without any evidence? The answer appears to be conflicts of interest: Many of the experts advising the WHO had financial ties to the pharmaceutical firms that produce the drugs (Cohen and Carter 2010). In this case, nudging helps to fill the bank accounts of the pharmaceutical industry and empty those of both the nudgers and the nudged.

#### PSA Tests

Sunstein (2005) proposed that “hospitals might frame options in a way that will lead people to choose medical procedures that are clearly best, even if a small probability of failure might frighten some patients and lead them to less promising options. There is a warning here about the popular idea of patient autonomy” (p. 180). Again, this would be a useful nudge if hospitals had no conflicting interests. Unfortunately, they often do and pursue goals diametrically opposed to those of their patients. For instance, many hospitals recommend that men take routine PSA tests for prostate cancer screening, despite the National Cancer Institute’s cautions that PSA screening can do more harm than good. By twisting health statistics in an advertisement, the highly respected U.S. cancer center MD Anderson systematically misled men about the benefits of prostate cancer screening, similar to how the pink ribbon organization Susan G. Komen deceived women about breast cancer screening (for details see Woloshin and Schwartz 2012; Woloshin, Schwartz, and Welch 2008).

As in the case of Tamiflu, access to the results of medical science is often impeded by a flood of biased information. For instance, a representative study in nine countries showed that more than 90 % of Europeans overestimate the benefits of PSA and mammography screening by a factor of 10, 100, 200, or do not know them (Gigerenzer, Mata, and Frank 2009). The reason is not that so many Europeans think irrationally; rather, they are “nudged” into screening by misleading statistics (Gigerenzer 2014a, b).

Similar conflicts of interest emerge when governments decide on the details for automatically enlisting people in pension plans (Rebonato 2012). Nudging may be effective if choice architects have the welfare of the public at heart. But in situations where politicians engage in defensive decisions, are risk illiterate, or have conflicting interests – the SIC Syndrome – nudging is not ecologically rational.

### A Word on “System 1” and “System 2”

Libertarian paternalists tend to explain all behavior with the help of two concepts of the mind. “System 1” is said to be fast and unconscious, to work by intuition and heuristics, and to be the cause of error and lack of rationality. In contrast, “System 2” is said to be slow and conscious, to work by logic and statistics, and to make no apparent errors. This vague distinction lacks formal precision of the underlying processes in each system; its only content is the assumption that these various dichotomies are aligned. But aligning heuristics with unconsciousness and errors is not correct (Kruglanski and Gigerenzer 2011). Every heuristic I have studied can be used both consciously and unconsciously, and in certain situations can lead to better performance than that of the logical or statistical models that are presented as rational per se. For instance, the 1/*N* heuristic is used consciously, not just unconsciously, by investors, and can make more money than mean-variance optimization *in investment situations where the heuristic is ecologically rational* (see above). One formal basis to understanding why and when less information is more beneficial is the bias-variance dilemma (Gigerenzer and Brighton 2009). Thus, heuristics, unconsciousness, and error proneness are not aligned in one system, as claimed. Nor are statistics, logic, and consciousness aligned in another system. As mentioned above, there is a long tradition in cognitive science that models unconscious or intuitive judgments by probability theory, inconsistent with Systems 1 and 2.

Where the real problem lies with the two-systems distinction is in its vagueness. It makes it possible to explain everything after the fact but not to deduce any interesting novel prediction. Usually, science progresses from vague dichotomies to precise models; the two-systems story is the only case I know of where it went the other way. Behavioral economists have reduced existing mathematical models of heuristic and statistical inference to two black boxes. Freud already had three systems.

## Conclusion: More Risk Savvy Citizens, Less Nudging

In this article, I assessed the scientific evidence presented for the justification of the nudging program: the claim that psychological research has clearly shown that people’s judgments systematically deviate from rationality and that it is extremely difficult, if not impossible, to educate people out of their biases. I focused on three alleged cognitive deviations from rationality: framing effects, base rate fallacy, and heuristics. My conclusion is that the justification of nudging rather than education assumes overly narrow logical norms of rationality and suffers from a confirmation bias, that is, selective reporting of relevant research. For each of the three “deviations,” the case for deviations from rationality is overstated, and evidence indicating that people are not educable is sparse.

Where does that leave us? As mentioned before, the true alternative to blaming and nudging people is to educate them. Being risk savvy encompasses both statistical thinking and heuristic thinking, and the awareness that the first applies to situations of risk, while the second is indispensable in situations of uncertainty. Usually, parts of the risks are known and others not, meaning that both of these tools are needed.

Libertarian paternalists believe that there is no alternative to their philosophy. But what if nudging stops when a different political party with other interests comes into power? What if the tobacco and fast-food industry counteracts by investing billions into nudging people into the opposite direction? Nudging people without educating them means infantilizing the public. This becomes particularly relevant in the digital revolution, as evident in Google’s search results, which were personalized in 2009. Since then, when two people perform the same search, they no longer see the same results. By providing personalized rank orders, search engines make us see what we like to see and what we have looked at before. This technique exploits the fact that about 90 % of the clicks are on the first page, with a third on the first result. Originally envisioned as a tool for providing the same information to every citizen on earth and creating a new era of transparency as the basis for democratic societies, the Internet now steers users into personalized bubbles where they are unlikely to encounter diverging points of view (Pariser, 2011). Manipulation of rank orders of search engine results about political candidates has been shown to influence the outcome of democratic elections, giving nudging a new political dimension (Epstein, R. and Roberson, R. E. The search engine manipulation effect (SEME): Search rankings can shift voter preferences substantially without their awareness (unpublshed)).

A more enduring solution in my view is investing in making people risk savvy. To be effective, education should start early, before young people are seduced into smoking, eating unhealthy food, and similar behaviors. I have sketched out such a program of becoming risk literate in health, finance, and digital media in Gigerenzer (2014b).

At issue is not simply the choice between paternalism and libertarianism. Nudging people into healthy behavior has limited chances of success when competing commercial firms with larger budgets use the same methods to nudge people into unhealthy behavior. Investing in risk-savvy citizens, by contrast, enables a sustainable solution: citizens who see through manipulation and can make informed decisions themselves.

## Footnotes

1 The confusion begins in Thaler and Sunstein’s (2008) book itself. First they clarify that a nudge is a tool that changes people’s behavior without “significantly changing their economic incentives” (p. 6)—after all, if nudges were incentives, they would be nothing new. But later, in their list of “a dozen nudges” (p. 232), the authors discuss the “dollar a day” program, in which city governments pay teenage girls who already have a baby a dollar for each day they are not pregnant. This program for reducing second pregnancies uses good old monetary incentives, which are now presented as nudges. Thaler and Sunstein comment: “Thus far, the results have been extremely promising” (p. 232) and cite a preliminary study with no control group. As an aside, I was curious about how big the effect was and in a literature search found one randomized trial by Stevens-Simon et al. (1997) with 286 girls. This controlled study reported that providing a dollar a day did not change the rates of second pregnancies at all.
